# Nurturing Resilient Minds in Nursing Students: Purpose in Life, Resilience, and Personality‐Based Substance Use Risk—A Descriptive Correlational Study

**DOI:** 10.1002/brb3.71898

**Published:** 2026-11-03

**Authors:** Mira Naguib Abdelrazek Elfar, Heba Mahmoud Mahmoud Mohamed, Shaimaa Mohamed Elghareeb Allam, Wafa Hamad Almegewly, Mazen Baazeem, Heba Mohammed Mahmoud Elhapashy, Mahmoud Abdelwahab Khedr

**Affiliations:** ^1^ Department of Psychiatric and Mental Health Nursing, Faculty of Nursing Alexandria University Alexandria Egypt; ^2^ Department of Nursing, College of Pharmacy, Nursing and Medical Sciences Riyadh Elm University Riyadh Saudi Arabia; ^3^ Medical Surgical Nursing Department, Faculty of Nursing Alexandria University Alexandria Egypt; ^4^ Medical‐Surgical Nursing Department Faculty of Nursing ‐ Mansoura University Mansoura Egypt; ^5^ Department of Community and Psychiatric Mental Health Nursing, College of Nursing Princess Nourah bint Abdulrahman University Riyadh Saudi Arabia; ^6^ Department of Health Information Management and Technology, College of Applied Medical Sciences University of Hafr Albatin Hafr Albatin Saudi Arabia; ^7^ College of Nursing University of Hafr Albatin Hafr Albatin Saudi Arabia

**Keywords:** nursing students, psychiatric nursing, positive mental health, resilience, substance use risk, life purpose

## Abstract

**Background:**

Nursing students face sustained academic and clinical stressors that may affect mental health and personality‐based substance use vulnerability. Evidence remains limited in Saudi and other Middle Eastern nursing‐student populations on whether purpose in life and resilience relate differently to the four substance use risk profile scale (SURPS) personality profiles. This distinction is relevant to profile‐specific prevention and student‐support services. The study examined relationships among a study‐specific modified Purpose in Life (PIL) score, resilience, and the four SURPS profiles and explored associations with demographic characteristics.

**Methods:**

We conducted a descriptive correlational study with 915 undergraduate nursing students at a university in Saudi Arabia using an online English‐language survey. The PIL test was administered in a study‐specific 20‐item, 5‐point online response format rather than the conventional 7‐point semantic‐differential format; consequently, the resulting PIL score was treated as a modified within‐sample measure and not as psychometrically equivalent to the conventional PIL. SURPS was scored as four separate personality profiles with the positively worded hopelessness items reverse‐scored, and CD‐RISC‐25 responses were recoded to the standard 0–4 metric before summation.

**Results:**

Mean scores were 60.55 ± 19.94 for the study‐specific modified PIL, 16.32 ± 4.49 for hopelessness, 13.06 ± 3.88 for anxiety sensitivity, 12.56 ± 3.75 for impulsivity, 15.36 ± 4.49 for sensation seeking, and 54.28 ± 24.60 for resilience. The modified PIL score correlated negatively with hopelessness (*r* = −0.763) and positively with resilience (*r* = 0.737); resilience correlated negatively with hopelessness (*r* = −0.770) and positively with anxiety sensitivity, impulsivity, and sensation seeking (*r* = 0.688, 0.612, and 0.683, respectively; all *p* < 0.001). Male students had slightly higher impulsivity and sensation‐seeking scores, while age and academic‐level differences were nonsignificant. Demographic models explained very little variance (*R* ^2^ = 0.006–0.012).

**Conclusion:**

Corrected SURPS scoring revealed a profile‐specific pattern, but the large correlations and the use of a nonvalidated 5‐point PIL response format require cautious interpretation. Hopelessness was strongly inversely associated with the modified PIL score and resilience, whereas anxiety sensitivity, impulsivity, and sensation seeking showed positive associations whose substantive meaning cannot be separated from possible construct overlap or shared‐method effects in this cross‐sectional self‐report dataset. The findings support separate SURPS‐profile interpretation rather than an aggregate score, while replication using the conventional PIL format, culturally validated instruments, direct behavioral outcomes, and formal construct‐validity testing is needed before stronger clinical inferences are made.

## Graphical abstract

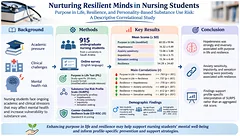

**Abbreviations**
CD‐RISC‐25: Connor‐Davidson Resilience Scale (25‐item version)
IRB: Institutional Review Board
PIL: Purpose in Life Test
SURPS: Substance Use Risk Profile Scale

## 1 Introduction

University nursing students experience sustained academic, clinical, and interpersonal demands that can affect psychological well‐being and increase vulnerability to maladaptive coping and substance‐related risk (McDermott et al. 2021; Martinez and Casaleiro 2022). Contemporary higher‐education guidance also emphasizes the need for integrated mental‐health and substance‐use support rather than symptom‐focused responses alone (American Psychological Association 2022; National Academies of Sciences, Engineering, and Medicine 2021). Saudi studies similarly document the importance of resilience and well‐being in nursing students during demanding academic and clinical training (Grande et al. 2021; Altharman et al. 2023; Rayani et al. 2024).

Positive mental health includes personal resources that support functioning, adaptation, and meaning (Silva et al. 2023). Resilience refers to the capacity to adapt to stressors and recover from adversity (Connor and Davidson 2003; Masten 2018), whereas purpose in life reflects meaning, direction, and life affirmation (Crumbaugh and Maholick 1964; Schulenberg et al. 2011). Studies of nursing students and young adults support links among resilience, psychological well‐being, self‐efficacy, and meaning in life (Arslan and Yıldırım 2021; Berdida et al. 2023; Rayani et al. 2024). In Saudi and Egyptian nursing students, resilience‐focused intervention has also improved related positive psychological outcomes, supporting the practical relevance of strengthening these resources (Khedr et al. 2023).

Personality‐based substance use vulnerability is commonly conceptualized through the Substance Use Risk Profile Scale (SURPS), which assesses hopelessness, anxiety sensitivity, impulsivity, and sensation seeking (Woicik et al. 2009). Validation studies support interpreting these as separate profiles rather than as a single global risk score (Schlauch et al. 2015; Long et al. 2018). Importantly, SURPS traits do not measure actual substance use. Regional evidence reinforces this distinction: among women at a Saudi university, specific SURPS traits were examined in relation to prescription‐opioid misuse, illustrating that trait profiles may be associated with behavior without being equivalent to behavior itself (Dabbagh et al. 2023).

The present conceptual model treats purpose in life and resilience as positive psychological resources while recognizing that the four SURPS domains represent different vulnerability pathways rather than a single negative continuum. Strong inverse relationships are most theoretically plausible for hopelessness because it directly reflects negative expectancy, whereas anxiety sensitivity, impulsivity, and sensation seeking can coexist with adaptive resources and therefore need not correlate in the same direction (Woicik et al. 2009; Ryff and Singer 2008; Wills and Dishion 2004). Examining the profiles separately is therefore necessary to avoid obscuring divergent associations.

Despite increasing evidence on nursing‐student resilience in Saudi Arabia and the Gulf (Grande et al. 2021; Grande et al. 2022; Altharman et al. 2023; Rayani et al. 2024), evidence remains limited on how purpose in life and resilience relate to each separate SURPS profile in Middle Eastern nursing students. This gap matters because aggregate risk interpretation could obscure profile‐specific patterns and lead to poorly targeted student‐support strategies. The study therefore addressed three questions: (1) What are the levels of purpose in life, resilience, and each SURPS personality profile? (2) How are purpose in life and resilience related to each SURPS profile? (3) Do these outcomes differ according to age, sex, or academic level?

## 2 Methods

### 2.1 Design

A descriptive correlational design was used to examine relationships among purpose in life, resilience, the four SURPS personality profiles, and selected demographic characteristics.

### 2.2 Setting

The study was conducted in an undergraduate nursing program at Riyadh Elm University in Saudi Arabia.

### 2.3 Participants and Sampling

The Epi Info program was used to estimate the minimum required sample size (*n* = 900) using a 5% margin of error, 95% confidence level, and 50% expected frequency. A total of 915 eligible nursing students completed the online survey and were included in the analysis. Recruitment was conducted electronically through the academic program. The archived recruitment records did not contain a denominator for the number of unique eligible students who received or opened the survey invitation; therefore, a conventional response rate could not be calculated. Verification of the source response dataset identified six Level 1 responses. For the four‐category academic‐level variable used in the revised analysis, these six Level 1 responses were combined with Level 2 in the lower academic‐level reference group, yielding *n* = 663 (72.5%); the remaining categories were Level 3 (*n* = 13, 1.4%), Level 4 (*n* = 208, 22.7%), and Level 5 (*n* = 31, 3.4%). The source documentation did not record the original rationale for this grouping or program‐level enrollment counts by sex and academic level; consequently, the representativeness of the observed sex and academic‐level distributions could not be assessed.

### 2.4 Eligibility Criteria

Students were eligible if they were currently enrolled as undergraduate nursing students and voluntarily agreed to participate. Students were excluded if they were not actively enrolled during the data collection period, declined participation, submitted incomplete questionnaires, or provided duplicate responses.

### 2.5 Instruments

Data were collected using a demographic questionnaire and three self‐report instruments: the Connor‐Davidson Resilience Scale (CD‐RISC‐25), the Substance Use Risk Profile Scale (SURPS), and the Purpose in Life Test (PIL). The instruments were administered in English. No study‐specific translation or cultural adaptation was undertaken, and formal cross‐cultural measurement equivalence was not tested in this Saudi nursing‐student sample. Accordingly, observed scores are interpreted as sample‐specific, and potential effects of language comprehension and cultural measurement non‐equivalence are considered in the interpretation of the findings.

#### 2.5.1 Connor‐Davidson Resilience Scale (CD‐RISC‐25)

The CD‐RISC‐25 is a 25‐item self‐report measure of resilience (Connor and Davidson 2003). The standard instrument uses five response options scored from 0 (not true at all) to 4 (true nearly all the time), producing a total score from 0 to 100, with higher scores indicating greater resilience. In the online export used for this study, the five response categories were numerically stored as 1–5; consequently, one point was subtracted from each item before the standard 0–4 item scores were summed. Internal consistency in the present sample was excellent (Cronbach's α = 0.977, 95% CI [0.975, 0.979]).

#### 2.5.2 Substance Use Risk Profile Scale (SURPS)

The SURPS is a 23‐item self‐report questionnaire assessing four personality‐based substance use risk profiles: hopelessness, anxiety sensitivity, impulsivity, and sensation seeking (Woicik et al. 2009). Items are rated on a 4‐point scale. The positively worded items within the hopelessness profile were reverse‐scored before calculating the hopelessness subscale. The four profiles were scored and analyzed separately; no overall SURPS total score was calculated or interpreted. Higher subscale scores indicate greater expression of the respective personality profile. Internal consistency coefficients were α = 0.774, 95% CI [0.751, 0.796] for hopelessness; α = 0.874, 95% CI [0.860, 0.886] for anxiety sensitivity; α = 0.837, 95% CI [0.819, 0.853] for impulsivity; and α = 0.856, 95% CI [0.841, 0.870] for sensation seeking. Hopelessness had the lowest reliability coefficient, indicating comparatively greater potential measurement error for this profile than for the other study measures.

#### 2.5.3 Purpose in Life Test (PIL)

The PIL test is a 20‐item measure of perceived meaning, goal direction, and existential fulfillment (Crumbaugh and Maholick 1964). The conventional PIL uses item‐specific 7‐point semantic‐differential responses. In the online form used in the present study, however, the 20 PIL items were administered using a harmonized 5‐point response format, with response coding oriented so that higher values represented greater purpose. Each item was presented with two descriptive response anchors representing lower and higher levels of intended purpose in life. Responses were recorded numerically from 1 to 5, with 1 corresponding to the lower‐purpose endpoint and 5 to the higher‐purpose endpoint. All 20 items were scored in the same direction, and no additional reverse scoring was applied during data analysis. Item scores were summed to a study‐specific total ranging from 20 to 100. Further details of the administered items, response directions, and scoring procedures are provided in Table S1. The exact questionnaire version, including the original item wording and response anchors, is retained by the research team and may be requested from the corresponding author, subject to applicable copyright permissions and institutional requirements.

This modification changes the response metric and was not formally validated; therefore, the resulting score is treated throughout this manuscript as a study‐specific modified PIL score rather than as psychometrically equivalent to the conventional PIL. Standard PIL cutoff values, normative interpretations, and direct score comparisons with studies using the original 7‐point format were not applied. Internal consistency was excellent (α = 0.968, 95% CI [0.965, 0.971]); however, high internal consistency does not establish validity of the modified response format. The modified PIL showed theoretically coherent associations with resilience (*r* = 0.737, 95% CI [0.706, 0.765]) and hopelessness (*r* = −0.763, 95% CI [−0.789, −0.735]), which provide limited nomological evidence only and should not be interpreted as formal validation or proof of construct distinctiveness.

### 2.6 Data Collection

Data were collected from March 2026 to May 2026 through an online English‐language questionnaire. After administrative permissions were obtained, eligible students were invited to participate electronically. The study's purpose, the voluntary nature of participation, confidentiality procedures, and right to withdraw were presented before the survey. Participants proceeded to the questionnaire after providing informed consent.

### 2.7 Ethical Considerations

The study received ethical approval from the Research Ethics Committee/Institutional Review Board at Riyadh Elm University (IRB No. FRP/2025/590/1388/1271). The approval was valid for 1 year beginning on March 1, 2026. Before enrollment, all participants received detailed information regarding the study's purpose, procedures, and requirements. Anonymity and confidentiality were assured throughout the research process, and all collected data were handled securely. Written informed consent was obtained before data collection. Participation was voluntary, and participants could withdraw at any time without penalty or adverse consequences. The study was conducted in accordance with the ethical principles of the 2024 revision of the Declaration of Helsinki (World Medical Association 2025) and established standards for research involving human participants.

### 2.8 Statistical Analysis

Data were analyzed using IBM SPSS Statistics version 26.0. Categorical variables were summarized using frequencies and percentages, and continuous variables using means and standard deviations. Independent‐samples t tests and one‐way analysis of variance (ANOVA) examined bivariate demographic differences. Pearson correlation coefficients examined relationships among the modified PIL score, the four SURPS profiles, and resilience; 95% confidence intervals for correlations were calculated using Fisher's z transformation. As a sensitivity analysis, Spearman rank correlations were also examined to assess whether correlation direction depended materially on distributional assumptions or extreme values. Internal consistency was assessed using Cronbach's α, with 95% confidence intervals calculated using the Feldt method. Multiple linear regression models were fitted separately for the modified PIL score, hopelessness, anxiety sensitivity, impulsivity, sensation seeking, and resilience to estimate adjusted demographic associations while considering age, sex, and academic level simultaneously. Age group and academic level were entered as categorical predictors using indicator variables; the reference categories were the youngest age group and the lower academic‐level group (Levels 1 and 2 combined). Sex was coded with male as the reference category. Statistical significance was set at *p* ≤ 0.05. Because the demographic models were exploratory, no formal multiplicity correction was imposed on the primary analyses; isolated coefficients were interpreted cautiously, and a conservative Bonferroni benchmark across six outcome models (α ≈ 0.0083) was considered in interpretation. *R* ^2^ and adjusted *R* ^2^ were used to evaluate explanatory power. Diagnostic reanalysis of the source dataset showed approximately symmetric composite‐score distributions (absolute skewness ≤ 0.20). Pearson correlations were directionally consistent with Spearman correlations, with a maximum absolute coefficient difference of 0.079. This sensitivity finding suggests that the unexpected correlation directions were not solely a consequence of rank‐order distortion or isolated extreme values, but it does not resolve possible construct overlap, shared‐method variance, or measurement non‐equivalence. Because all regression predictors were categorical and indicator‐coded, a continuous‐predictor linearity assumption was not applicable. Regression residuals were approximately symmetric (skewness −0.197 to 0.080). Breusch‐Pagan tests were nonsignificant for five of the six models (*p* = 0.100–0.322); the modified PIL model showed mild heteroscedasticity (*p* = 0.045), and an HC3‐robust standard‐error sensitivity analysis did not alter its nonsignificant interpretation. Statistical notation was standardized throughout the Abstract, Results, and tables: *r*, α, β, *R* ^2^, and *p* values are reported without a leading zero; correlation coefficients and exact p values are reported to three decimal places, with *p* < 0.001 used for smaller values.

## 3 Results

### 3.1 Sample Characteristics

A total of 915 undergraduate nursing students were included in the analysis. Most participants were aged 21–25 years (63.6%), followed by those aged 18–20 years (30.3%) and those older than 25 years (6.1%). The sample was predominantly female (73.9%). For the academic‐level variable, participants were classified into four categories: Levels 1–2 combined (*n* = 663, 72.5%), Level 3 (*n* = 13, 1.4%), Level 4 (*n* = 208, 22.7%), and Level 5 (*n* = 31, 3.4%). The lower academic‐level group included six Level 1 students and 657 Level 2 students. This classification was used consistently throughout the analyses (Table 1).

**TABLE 1:** Demographic characteristics of the study sample (*n* = 915).

Characteristic	No.	%
Age (years)		
18–20	277	30.3
21–25	582	63.6
>25	56	6.1
Sex		
Male	239	26.1
Female	676	73.9
Academic level		
Levels 1–2	663	72.5
Lvel third	13	1.4
Level fourth	208	22.7
Level fifth	31	3.4

*Note*: Nationality has been removed because it was not present in the source response dataset used for reanalysis. Age categories were confirmed as 18–20 years, 21–25 years, and >25 years. The lower academic‐level category combines six Level 1 responses with Level 2 responses (*n* = 663, 72.5%).

### 3.2 Descriptive Scores for Purpose in Life, SURPS Profiles, and Resilience

All results reported below use the corrected SURPS scoring, including reverse‐scoring of the positively worded hopelessness items; this item‐level correction materially changed the direction of the hopelessness associations. As shown in Table 2, the mean study‐specific PIL score was 60.55 ± 19.94, and the mean standard‐scored CD‐RISC‐25 total was 54.28 ± 24.60. The corrected SURPS subscale means were 16.32 ± 4.49 for hopelessness, 13.06 ± 3.88 for anxiety sensitivity, 12.56 ± 3.75 for impulsivity, and 15.36 ± 4.49 for sensation seeking. No overall SURPS total was calculated. Standard PIL cutoff categories were not used because the online PIL response format differed from the conventional 7‐point format.

**TABLE 2:** Descriptive scores and internal consistency of the study variables (*n* = 915).

Variable	Scoring range	Mean ± SD	Cronbach's α
Purpose in Life Test (PIL), study‐specific 5‐point format	20–100	60.55 ± 19.94	0.968
SURPS—Hopelessness	7–28	16.32 ± 4.49	0.774
SURPS—Anxiety sensitivity	5–20	13.06 ± 3.88	0.874
SURPS—Impulsivity	5–20	12.56 ± 3.75	0.837
SURPS—Sensation seeking	6–24	15.36 ± 4.49	0.856
Connor‐Davidson Resilience Scale (CD‐RISC‐25)	0–100	54.28 ± 24.60	0.977

*Note*: SURPS profiles are reported separately; no overall SURPS total score is used. Positively worded hopelessness items were reverse‐scored. CD‐RISC‐25 responses exported as 1–5 were recoded to the standard 0–4 metric. The PIL was administered using a study‐specific 5‐point response format rather than the conventional 7‐point format; therefore, conventional PIL cutoffs and direct normative comparisons are not applicable.

### 3.3 Correlations Among Study Variables

Table 3 presents the corrected Pearson correlations. The modified PIL score was negatively correlated with hopelessness (*r* = −0.763, 95% CI [−0.789, −0.735]) and positively correlated with anxiety sensitivity (*r* = 0.659, 95% CI [0.621, 0.694]), impulsivity (r = 0.592, 95% CI [0.548, 0.633]), sensation seeking (*r* = 0.665, 95% CI [0.627, 0.700]), and resilience (*r* = 0.737, 95% CI [0.706, 0.765]). Resilience was negatively correlated with hopelessness (*r* = −0.770, 95% CI [−0.795, −0.742]) and positively correlated with anxiety sensitivity (*r* = 0.688, 95% CI [0.652, 0.721]), impulsivity (*r* = 0.612, 95% CI [0.570, 0.651]), and sensation seeking (*r* = 0.683, 95% CI [0.647, 0.716]). Hopelessness was negatively related to the other three SURPS profiles (*r* = −0.625 to −0.736), whereas anxiety sensitivity, impulsivity, and sensation seeking were strongly positively intercorrelated (*r* = 0.810–0.831). All reported Pearson correlations were statistically significant at *p* < 0.001. In the sensitivity analysis, Spearman correlations retained the same direction as the Pearson coefficients, and the largest absolute difference between corresponding coefficients was 0.079. Thus, the unexpected positive directions were not reversed by rank‐based analysis, although this does not exclude construct overlap or shared‐method effects.

**TABLE 3:** Pearson correlation matrix among study variables (*n* = 915).

Variable	PIL	H	AS	IMP	SS	Resilience
PIL	—					
H	−0.763*	—				
AS	0.659*	−0.736*	—			
IMP	0.592*	−0.625*	0.810*	—		
SS	0.665*	−0.713*	0.812*	0.831*	—	
Resilience	0.737*	−0.770*	0.688*	0.612*	0.683*	—

*Note*: PIL = study‐specific modified Purpose in Life score; H = Hopelessness; AS = Anxiety sensitivity; IMP = Impulsivity; SS = Sensation seeking. Correlation coefficients are reported to three decimal places. *p < 0.001 for all reported correlations.

### 3.4 Associations With Demographic Characteristics

Differences in the six study outcomes according to demographic characteristics are presented in Table 4. No statistically significant age‐group differences were observed for purpose in life, any SURPS profile, or resilience (all *p* ≥ 0.107). Male students had higher impulsivity scores than female students (13.08 ± 3.55 vs. 12.37 ± 3.81, *p* = 0.013) and higher sensation‐seeking scores (15.99 ± 4.20 vs. 15.14 ± 4.57, *p* = 0.012); the other sex comparisons were not significant. No statistically significant differences were identified across the four academic‐level groups for any outcome (all *p* ≥ 0.242).

**TABLE 4:** Relationships between demographic characteristics and study outcomes (*n* = 915).

Characteristic	PIL mean ± SD	H mean ± SD	AS mean ± SD	IMP mean ± SD	SS mean ± SD	Resilience mean ± SD
Age (years)						
18–20	59.79 ± 20.85	16.43 ± 4.60	13.04 ± 4.08	12.55 ± 3.95	15.28 ± 4.71	53.48 ± 24.96
21–25	60.40 ± 19.28	16.36 ± 4.44	12.98 ± 3.77	12.50 ± 3.66	15.29 ± 4.38	54.07 ± 24.18
>25	65.89 ± 21.57	15.41 ± 4.49	13.96 ± 3.96	13.25 ± 3.72	16.48 ± 4.41	60.43 ± 26.66
*F*(*p*)	*F* = 2.236; *p* = 0.107	*F* = 1.255; *p* = 0.286	*F* = 1.649; *p* = 0.193	*F* = 1.031; *p* = 0.357	*F* = 1.868; *p* = 0.155	*F* = 1.920; *p* = 0.147
Sex						
Male	61.74 ± 18.50	16.11 ± 4.26	13.42 ± 3.63	13.08 ± 3.55	15.99 ± 4.20	55.66 ± 22.78
Female	60.13 ± 20.42	16.40 ± 4.57	12.93 ± 3.96	12.37 ± 3.81	15.14 ± 4.57	53.79 ± 25.21
*t*(*p*)	*t* = 1.077; *p* = 0.282	*t* = ‐0.843; *p* = 0.399	*t* = 1.692; *p* = 0.091	*t* = 2.488; *p* = 0.013	*t* = 2.520; *p* = 0.012	*t* = 1.008; *p* = 0.313
Academic level						
Levels 1 and 2	60.14 ± 20.06	16.41 ± 4.53	13.03 ± 3.95	12.47 ± 3.78	15.27 ± 4.53	53.77 ± 24.94
Level third	60.31 ± 8.34	17.77 ± 2.98	11.69 ± 3.12	11.77 ± 2.77	15.38 ± 3.88	47.92 ± 12.76
Level fourth	61.08 ± 20.06	16.12 ± 4.39	13.16 ± 3.67	12.74 ± 3.70	15.40 ± 4.33	55.22 ± 23.70
Level fifth	65.97 ± 19.82	15.29 ± 4.73	13.61 ± 3.91	13.55 ± 3.90	16.94 ± 4.65	61.58 ± 26.15
*F*(*p*)	*F* = 0.906; *p* = 0.437	*F* = 1.206; *p* = 0.306	*F* = 0.814; *p* = 0.486	*F* = 1.186; *p* = 0.314	*F* = 1.362; *p* = 0.253	*F* = 1.398; *p* = 0.242

*Note*: *t* = independent‐samples *t* test; *F* = one‐way ANOVA. The lower academic‐level category combines Levels 1 and 2. Only the sex differences for impulsivity (*p* = 0.013) and sensation seeking (*p* = 0.012) were statistically significant at *p* ≤ 0.05.

### 3.5 Regression Analyses

Table 5 presents the re‐estimated multivariable regression models using categorical indicator coding for age group and academic level and separate SURPS profile outcomes. All models had very low explanatory power (*R* ^2^ = 0.006–0.012; adjusted *R* ^2^ approximately 0.000–0.005), and none of the overall model F tests reached statistical significance (*p* = 0.094–0.431). Female sex showed small negative coefficients for impulsivity (B = −0.660, β = −0.077, *p* = 0.023) and sensation seeking (B = −0.745, β = −0.073, *p* = 0.031); no other demographic coefficient was statistically significant. These isolated coefficients would not meet a conservative Bonferroni benchmark of α ≈ 0.0083 across six outcome models. Given the nonsignificant overall models, minimal explained variance, and multiplicity of exploratory tests, these coefficients should be interpreted cautiously.

**TABLE 5:** Multiple linear regression analyses for demographic correlates of study outcomes (*n* = 915).

Outcome	Predictor	B	β	*t*	*p*	95% CI	Model fit
Purpose in Life	Age 21–25	0.710	0.017	0.485	0.628	−2.163 to 3.583	*R* ^2^ = 0.007; adj *R* ^2^ = 0.001; *F* = 1.086; *p* = 0.369
	Age >25	5.315	0.064	1.748	0.081	−0.653 to 11.283	
	Female	−1.223	−0.027	−0.796	0.426	−4.239 to 1.793	
	Academic third	−0.597	−0.004	−0.106	0.915	−11.602 to 10.408	
	Academic fourth	1.067	0.022	0.673	0.501	−2.045 to 4.179	
	Academic fifth	3.528	0.032	0.916	0.360	−4.033 to 11.090	
Hopelessness	Age 21–25	−0.078	−0.008	−0.238	0.812	−0.726 to 0.569	*R* ^2^ = 0.006; adj *R* ^2^ = −0.000; *F* = 0.989; *p* = 0.431
	Age >25	−0.897	−0.048	−1.309	0.191	−2.241 to 0.448	
	Female	0.239	0.023	0.691	0.490	−0.440 to 0.919	
	Academic third	1.510	0.040	1.195	0.232	−0.969 to 3.990	
	Academic fourth	−0.303	−0.028	−0.847	0.397	−1.004 to 0.398	
	Academic fifth	−0.706	−0.028	−0.813	0.416	−2.410 to 0.998	
Anxiety sensitivity	Age 21–25	−0.020	−0.002	−0.069	0.945	−0.578 to 0.539	*R* ^2^ = 0.009; adj *R* ^2^ = 0.002; *F* = 1.334; *p* = 0.239
	Age >25	0.899	0.056	1.520	0.129	−0.261 to 2.059	
	Female	−0.472	−0.054	−1.581	0.114	−1.059 to 0.114	
	Academic third	−1.566	−0.048	−1.436	0.151	−3.706 to 0.574	
	Academic fourth	0.164	0.018	0.533	0.594	−0.441 to 0.769	
	Academic fifth	0.065	0.003	0.087	0.931	−1.405 to 1.536	
Impulsivity	Age 21–25	0.006	0.001	0.023	0.982	−0.534 to 0.546	*R* ^2^ = 0.011; adj *R* ^2^ = 0.004; *F* = 1.683; *p* = 0.122
	Age >25	0.534	0.034	0.934	0.350	−0.587 to 1.655	
	Female	−0.660	−0.077	−2.286	0.023	−1.227 to −0.093	
	Academic third	−0.946	−0.030	−0.898	0.370	−3.014 to 1.122	
	Academic fourth	0.290	0.032	0.972	0.331	−0.295 to 0.874	
	Academic fifth	0.621	0.030	0.858	0.391	−0.800 to 2.042	
Sensation Seeking	Age 21–25	0.071	0.008	0.217	0.828	−0.574 to 0.716	*R* ^2^ = 0.012; adj *R* ^2^ = 0.005; *F* = 1.810; *p* = 0.094
	Age >25	0.896	0.048	1.312	0.190	−0.444 to 2.235	
	Female	−0.745	−0.073	−2.161	0.031	−1.422 to −0.068	
	Academic third	−0.184	−0.005	−0.146	0.884	−2.655 to 2.286	
	Academic fourth	0.158	0.015	0.444	0.657	−0.541 to 0.856	
	Academic fifth	1.050	0.042	1.214	0.225	−0.648 to 2.747	
Resilience	Age 21–25	0.652	0.013	0.361	0.718	−2.891 to 4.195	*R* ^2^ = 0.008; adj *R* ^2^ = 0.002; *F* = 1.238; *p* = 0.284
	Age >25	5.936	0.058	1.583	0.114	−1.422 to 13.295	
	Female	−1.466	−0.026	−0.774	0.439	−5.185 to 2.253	
	Academic third	−6.760	−0.033	−0.978	0.328	−20.330 to 6.810	
	Academic fourth	1.594	0.027	0.815	0.415	−2.243 to 5.431	
	Academic fifth	5.171	0.038	1.089	0.277	−4.152 to 14.495	

*Note*: Reference categories: age 18–20 years, male sex, and lower academic level (Levels 1 and 2 combined). Age group and academic level were represented by indicator (dummy) variables. β = standardized coefficient; CI = confidence interval. *p*‐values are reported to three decimal places. All overall models had minimal explanatory power and were not statistically significant.

## 4 Discussion

### 4.1 Principal Findings

The item‐level re‐audit identified a scoring error in the SURPS hopelessness profile: positively worded hopelessness items had not been reverse‐scored in the original analysis. After correction, the modified PIL score and resilience were strongly and inversely related to hopelessness, whereas anxiety sensitivity, impulsivity, and sensation seeking remained positively associated with both positive psychological‐resource measures. Demographic associations were weak, and all regression models explained less than 1.2% of outcome variance. Because several correlations were large and some directions were counterintuitive, these findings are interpreted as sample‐specific associations rather than evidence of protective effects or distinct causal pathways. The results support separate SURPS‐profile scoring, but they also raise measurement questions that require formal construct‐validity testing in future work.

### 4.2 Comparison With Prior Empirical Literature

The strong positive association between the modified PIL score and resilience is consistent in direction with prior evidence linking meaning in life with resilience and mental well‐being (Arslan and Yıldırım 2021) and with nursing‐student research showing that resilience covaries with psychological well‐being and other positive resources (Grande et al. 2021; Berdida et al. 2023; Rayani et al. 2024). The inverse pattern for hopelessness is likewise conceptually coherent with the contrast between negative expectancy and positive psychological functioning. By contrast, the positive associations of anxiety sensitivity, impulsivity, and sensation seeking with the modified PIL score and resilience are counterintuitive and should not be interpreted as evidence that these traits are beneficial or protective against substance use. In a Saudi university sample, Dabbagh et al. (2023) linked specific SURPS traits to prescription‐opioid misuse, underscoring that personality vulnerability and positive psychological resources are conceptually distinct from actual substance‐use behavior. The present data cannot determine whether the positive associations represent genuine co‐expression of traits in this sample, overlap among measured constructs, response‐pattern effects, or another measurement artifact; therefore, substantive interpretation of these directions is deliberately restrained.

### 4.3 Magnitude, Construct Overlap, and Discriminant Validity

Several observed correlations were large (approximately |*r*| = 0.59–0.77), corresponding to roughly 35%–59% shared variance for the principal associations involving the modified PIL score and resilience. The concern is stronger within the non‐hopelessness SURPS profiles: anxiety sensitivity, impulsivity, and sensation seeking correlated at *r* = 0.810–0.831, equivalent to approximately 66%–69% shared variance. Such magnitudes do not establish that the constructs are interchangeable, but they raise a substantive discriminant‐validity concern and increase the plausibility of construct overlap or shared‐method variance. The modified PIL–CD‐RISC‐25 correlation (*r* = 0.737) is also high enough to require caution because both measures contain content related to direction, adaptation, confidence, and psychological functioning. Contemporary measurement guidance recommends evaluating discriminant validity rather than assuming it from scale labels alone (Rönkkö and Cho 2022). Formal confirmatory factor analysis, heterotrait‐monotrait assessment, or latent method‐factor analysis was not performed in this study. Consequently, the present data should not be taken as evidence that these constructs are empirically distinct; the correlations are interpreted only as associations among observed scale scores in this sample, and formal construct‐validity testing is required in future psychometric work. Formal HTMT assessment or confirmatory factor analysis was not added to the present study because the study was designed as a descriptive correlational investigation rather than a psychometric validation study, and a joint latent measurement model was not prespecified. In addition, the PIL was administered using a study‐specific, nonvalidated 5‐point response format and the instruments were used in English without formal cross‐cultural measurement‐equivalence testing in this population. Under these conditions, a post hoc CFA or HTMT analysis could be overinterpreted as establishing construct validity beyond what the study design and measurement conditions support. We therefore treat discriminant validity as unresolved and the observed high correlations as a signal requiring dedicated, item‐level psychometric evaluation with a prespecified measurement model in future validation work.

### 4.4 Demographic and Regional Context

Age and academic level were not significantly associated with the six outcomes, and sex differences were limited to small differences in impulsivity and sensation seeking. The higher sensation‐seeking scores among men are consistent with meta‐analytic evidence that men tend to report higher sensation seeking (Cross et al. 2013), although the present sex effects were small and the regression models were nonsignificant overall. The null age and academic‐level findings are also compatible with Saudi nursing‐student research reporting no clear age, gender, or year‐level differences in resilience and well‐being (Grande et al. 2021) and with a Saudi structural‐equation study in which profile variables were not associated with academic resilience or quality of life (Grande et al. 2022). Other Saudi evidence has found some differences between junior and senior students (Rayani et al. 2024), suggesting that demographic patterns are not uniform across samples. In the present study, the narrow age distribution, female predominance, and heavy concentration in the second academic‐level group may have reduced between‐group variability and the ability to detect demographic associations. The Saudi setting is therefore an important contextual contribution, but cultural, service‐access, and stigma‐related mechanisms were not directly measured and should not be inferred from these data.

### 4.5 Implications for Psychiatric‐Mental Health Nursing and University Policy

University mental‐health services should not treat SURPS as a single aggregate risk score or use personality profiles as proxies for actual substance use. Given the measurement and design limitations of the present study, the findings are not sufficient to justify profile‐based clinical screening on their own. A cautious application is to maintain broadly accessible resilience‐ and purpose‐supportive programming while reserving confidential, profile‐specific assessment for contexts in which validated instruments, direct assessment of substance‐use behavior, and clear referral pathways are available. Resilience‐focused and mindfulness‐oriented interventions have shown promise for positive psychological outcomes in university and nursing‐student populations (Khedr et al. 2023; González‐Martín et al. 2023). Because demographic predictors were weak here, the present data do not support restricting access primarily by age, sex, or academic level. Any profile‐informed prevention strategy should be prospectively evaluated before routine implementation.

### 4.6 Contributions and Limitations

**Contributions**: This study provides a profile‐specific examination of a study‐specific modified PIL score, resilience, and personality‐based substance‐use vulnerability in an under‐studied Saudi/Middle Eastern nursing‐student context. The item‐level scoring re‐audit is a methodological contribution because it demonstrates how failure to reverse‐score positively worded hopelessness items can materially change substantive conclusions. The study also makes transparent an important psychometric caution: several observed associations were sufficiently large to raise questions about construct distinctiveness. Accordingly, the main contribution is descriptive and hypothesis‐generating rather than confirmatory. The findings reinforce the importance of scoring SURPS profiles separately while avoiding inference from personality profiles to actual substance‐use behavior.

**Limitations**: The cross‐sectional design precludes causal or temporal inference, and all measures were self‐reported in the same survey, creating potential social‐desirability, acquiescence, and shared‐method effects. The study assessed personality‐based substance‐use risk profiles rather than actual substance use, so the observed SURPS associations cannot be interpreted as associations with substance‐use behavior. Several correlations were large, including strong intercorrelations among anxiety sensitivity, impulsivity, and sensation seeking, and formal discriminant‐validity testing was not performed; therefore, construct overlap or a measurement artifact cannot be excluded. The survey was administered in English without study‐specific cultural adaptation or testing of cross‐cultural measurement equivalence, which may have influenced item interpretation. The sample was predominantly female (73.9%) and heavily concentrated in the lower academic‐level reference group that combined Levels 1 and 2 (72.5%), limiting subgroup balance and potentially reducing demographic precision. Archived recruitment records did not retain the number of unique eligible students who received or opened the invitation, so a conventional response rate could not be calculated and nonresponse bias cannot be quantified. Source‐data reanalysis clarified the academic‐level coding, but the archived documentation did not preserve the original rationale for combining Levels 1 and 2 or establish whether the observed sample distribution mirrored underlying program enrollment. Diagnostic reanalysis found mild heteroscedasticity in the purpose‐in‐life regression model, although HC3‐robust sensitivity analysis did not change its nonsignificant interpretation. Most importantly, the PIL was administered using a study‐specific 5‐point response format rather than its conventional 7‐point semantic‐differential format, and that adaptation was not formally validated. Thus, the modified PIL score should be interpreted only within this sample; conventional cutoffs, normative classification, direct score comparability, and claims of psychometric equivalence are not warranted. Future multicenter studies should use the conventional validated PIL format or formally validate any adaptation, employ culturally and linguistically validated instruments, retain complete recruitment documentation, test construct distinctiveness with factor‐analytic and discriminant‐validity methods, and directly measure substance‐use behavior when that is the outcome of interest.

## 5 Conclusion

After corrected SURPS scoring, hopelessness was strongly inversely associated with the study‐specific modified PIL score and resilience, whereas anxiety sensitivity, impulsivity, and sensation seeking showed positive associations. Because the PIL response format was modified without formal validation and several correlations were large, these associations should be regarded as exploratory and sample‐specific; construct overlap, shared‐method effects, or other measurement artifacts cannot be excluded. The findings support analyzing SURPS profiles separately and caution against inferring actual substance use from personality scores. They do not, however, establish a basis for profile‐driven clinical screening. Replication in multicenter samples using conventional or formally validated PIL scoring, culturally validated instruments, direct behavioral outcomes, and formal construct‐validity testing is needed before stronger practical or causal conclusions are drawn.

## Author Contributions

M.N.A.E. and H.M.M.M. conceptualized the study, developed the research design, and supervised data collection. W.H.A. contributed to methodology development, data interpretation, and critical revision of the manuscript. M.B. and H.M.M.E. participated in data analysis, interpretation of findings, and manuscript review. M.A.K. and S.M.E.A. contributed to study conception, statistical analysis, interpretation of results, manuscript drafting, and overall project supervision. All authors participated in reviewing and editing the manuscript, approved the final version for publication, and agree to be accountable for all aspects of the work.

## Funding

This research was funded by Princess Nourah Bint Abdulrahman University through the Researchers Supporting Project (PNURSP2026R312), Riyadh, Saudi Arabia.

## Ethics Statement

Ethical approval and consent procedures are described in Section 2.7 (Riyadh Elm University IRB No. FRP/2025/590/1388/1271). The study was conducted in accordance with the 2024 revision of the Declaration of Helsinki (World Medical Association 2025).

## Conflicts of Interest

The authors declare no conflicts of interest.

## Supporting information




**Supplementary Material**: brb371898‐sup‐0001‐TableS1.pdf

## Data Availability

The de‐identified dataset and the scoring codebook used for the revised analyses may be made available from the corresponding author on reasonable request, subject to institutional and ethics requirements.
