# Supplementary material for: Nurturing Resilient Minds in Nursing Students: Purpose in Life, Resilience, and Personality‐Based Substance Use Risk—A Descriptive Correlational Study
Source: Brain Behav. 2026 Nov 3;16(11):e71898. doi: 10.1002/brb3.71898 (PMC13729826; doi:10.1002/brb3.71898)
Supplement: Supplementary file 1 [file BRB3-16-e71898-s001.pdf]

The Purpose in Life Test (PIL)

(Crumbaugh & Maholick, 1964)

Write the number (1 to 5) next to each statement that is <u>most true</u> for you right now.

Items	Rate each item from 1 to 7	
**1. I am usually:**	bored<br>**1 ---------------2---------------3---------------4---------------5**	enthusiastic
**2. Life seems to me:**	completely routine;	always exciting.
**3. In life I have:**	no goals or aims;	clear goals and aims.
**4. My personal existence is:**	utterly meaningless, without purpose	purposeful and meaningful.
**5. Every day is:**	exactly the same;	constantly new and different.
**6. If I could choose, I would:**	prefer to never have been born;	want 9 more lives just like this one.
**7. After retiring, I would:**	loaf completely the rest of my life	do some of the exciting things I've always wanted to
**8. In achieving life goals I've:**	made no progress whatever	progressed to complete fulfillment
**9. My life is:**	empty, filled only with despair	running over with exciting things
**10. If I should die today, I'd feel that my life has been:**	completely worthless	very worthwhile
**11. In thinking of my life, I:**	often wonder why I exist	always see reasons for being here
**12. As I view the world in relation to my life, the world:**	completely confuses me	fits meaningfully with my life
**13. I am a:**	very irresponsible person	very responsible person
**14. Concerning freedom to choose, I believe humans are:**	completely bound by limitations of heredity and environment	totally free to make all life choices
**15. With regard to death, I am:**	unprepared and frightened	prepared and unafraid
**16. Regarding suicide, I have:**	thought of it seriously as a way out	never given it a second thought
**17. I regard my ability to find a purpose or mission in life as:**	practically none	very great
**18. My life is:**	out of my hands and controlled by external factors	in my hands and I'm in control of it
**19. Facing my daily tasks is:**	a painful and boring experience	a source of pleasure and satisfaction
**20. I have discovered:**	no mission or purpose in life	a satisfying life purpose

Scoring: Add up all the scores for each item (20-100). A score of less than 50 may indicate that you are experiencing significant "existential concerns" in your life.*

* This instrument is only provided here as a way of exploring one's responses to these different existential questions. I am not in any way advocating for its clinical utility or its ability to provide any credible diagnostic value. Please be aware, for example, that the conceptual validity of this scale and some of its individual items has been questioned (e.g. high scores may be indicative of depression, not necessarily existential issues). In addition, there is significant cultural variability in responses – it may not be appropriate for culturally diverse populations. Please use it as a tool to 'think about' these issues and nothing more.

©Brad Peters
www.bpeters.ca
